# Effects of real-time visual feedback (D-WALL) on neuromuscular control in healthy adults: an fNIRS study

**DOI:** 10.3389/fnhum.2026.1902433

**Published:** 2026-09-03

**Authors:** Yuli Shuai, Tianfeng Ye, Shunyuan Liu, Yizhan Wang, Liu Ye, Dandong Wu

**Affiliations:** 1 Department of Rehabilitation Medicine, Key Laboratory of Physical Medicine and Precision Rehabilitation of Chongqing Municipal Health Commission, The First Affiliated Hospital of Chongqing Medical University, Chongqing, China; 2 Department of Rehabilitation Medicine, Jinzishan Campus, Chongqing Mental Health Center, Chongqing, China

**Keywords:** functional near-infrared spectroscopy, healthy adults, postural neuromuscular control, surface electromyography, visual feedback

## Abstract

**Background:**

Neuromuscular control is essential for maintaining posture and performing motor tasks. Real-time visual feedback provides sensory information that may influence central neural processing and lower-limb muscle coordination during balance tasks. This study investigated the effects of visual feedback on neuromuscular control in healthy adults.

**Methods:**

Thirty healthy adults aged 18–40 years completed four randomized balance conditions with or without real-time visual feedback. Physical activity and sports participation were assessed using the Tegner Activity Scale. Static and dynamic balance asymmetry were evaluated using the Prokin Balance System and Y-Balance Test, respectively. Prefrontal cortical activity was recorded using functional near-infrared spectroscopy (fNIRS), and lower-limb muscle activation was assessed using surface electromyography (sEMG).

**Results:**

Among participants with lower physical activity levels (Tegner score < 5), left prefrontal cortex (lPFC) activation was significantly higher with visual feedback than without feedback. Among participants with greater bilateral balance asymmetry, right prefrontal cortex (rPFC) activation was significantly higher during cross-body X-shaped arm movements performed with visual feedback. Exploratory sEMG data from seven eligible participants further showed a higher relative activation ratio of the left posterior to anterior thigh muscles during visual-feedback tasks in participants with lower physical activity and poorer balance symmetry.

**Conclusion:**

Visual feedback was associated with altered prefrontal activation and reorganization of lower-limb muscle coordination during motor tasks in healthy adults.

## 1 Background

Neuromuscular control is fundamental to postural stability and motor performance, including movement speed, jumping, and agility. It depends on coordinated sensory input, central neural processing, and motor output (Trotman et al., 2025). Proprioceptive, vestibular, and visual signals contribute to immediate postural responses and are relayed to the motor cortex and brainstem to regulate muscle coordination (Trotman et al., 2025; Rasman et al., 2018). Adequate neuromuscular fitness may therefore improve motor performance and reduce injury risk (Lloyd et al., 2014).

Visual feedback has increasingly been incorporated into movement training in sports and rehabilitation. By presenting biomechanical information in real time, such as cursor-based changes in joint position and muscle responses, it enables individuals to monitor and adjust their own movements (Kasuga et al., 2022). This approach may complement conventional instructor-led training. In patients with stroke, real-time visual feedback combined with sit-to-stand training has improved strength, balance, gait, and quality of life (Hyun et al., 2021). Its accessibility also supports potential use in home-based rehabilitation (Moffet et al., 2017).

Visual information is integrated within neural pathways that coordinate movement (Du et al., 2026). During sensorimotor practice, real-time visual cues may promote sensorimotor learning, as supported by neuroimaging studies. In a video game-based balance task, superior temporal gyrus activation varied with task difficulty (Karim et al., 2012), while supplementary visual feedback during goal-directed tasks increased cortical activity (Wasaka et al., 2022). Visual contributions to postural control also show hemispheric asymmetry, with a predominant role of the right hemisphere in healthy adults (Pérennou et al., 1997).

Central nervous system activity can be assessed using positron emission tomography, functional magnetic resonance imaging, electroencephalography, and other techniques (Chollet et al., 1991; Cramer et al., 1997). Functional near-infrared spectroscopy (fNIRS) is particularly suitable for movement studies because it measures cortical changes in oxygenated hemoglobin (HbO_2_) and deoxygenated hemoglobin (HbR) during task performance (Yang and Wang, 2025; Verghese et al., 2017; Carrera and Tononi, 2014). It has therefore been widely used to record neural activity during motor tasks in healthy individuals (Bishnoi and Holtzer, 2021; Pelicioni et al., 2019; Miyai et al., 2001; Kurz et al., 2012; Kim et al., 2016).

The prefrontal cortex (PFC) is activated during postural stabilization in response to external perturbations (Xu et al., 2024). Although fNIRS and combined fNIRS–surface electromyography (sEMG) have been used to examine motor tasks, most studies have focused on virtual reality or mirror-therapy paradigms; comparatively few have investigated real-time visual feedback training (Pelicioni et al., 2019; Inagaki et al., 2019; Krelove et al., 2025). We hypothesized that visual feedback would be associated with greater task-related PFC activation and that this response would vary with postural balance performance.

## 2 Methods

### 2.1 Participants

Healthy adults aged 18–40 years were recruited between February and July 2025. Exclusion criteria were a history of lower-limb injury or surgery; neurological or vascular disorders affecting balance; ankle or foot pain or swelling; and visual or vestibular impairment. All participants received information about the study objectives, procedures, and precautions and provided written informed consent before enrollment. The study was approved by the Research Ethics Committee of the First Affiliated Hospital of Chongqing Medical University and registered in the Chinese Clinical Trial Registry (ChiCTR2500109898).

Before testing, participants completed the Edinburgh Handedness Inventory to determine limb dominance (Oldfield, 1971). The inventory assesses preference across 10 everyday tasks. A laterality quotient was calculated from the responses, with positive values indicating right-handedness.

### 2.2 Balance assessments

#### 2.2.1 Static balance

The Prokin Balance System (TecnoBody S.r.l., Italy) uses a force platform to quantify postural stability from center-of-pressure (CoP) displacement (Ke et al., 2022). Participants stood on the platform with their feet 10 cm apart and arms relaxed at their sides while the screen displayed real-time feedback of the CoP trajectory. Each participant completed a 30-s, eyes-open, single-leg standing trial on each limb in a quiet, controlled environment. Two trials per limb were averaged to improve measurement reliability.

#### 2.2.2 Dynamic balance

The Y-Balance Test (YBT) evaluates dynamic lower-limb function and joint stability. During single-leg stance, participants reached as far as possible with the contralateral limb in the anterior, posteromedial, and posterolateral directions. Each direction was tested three times, and the maximum reach distance was retained. Reach distances were normalized using standard procedures to allow within- and between-participant comparisons.

#### 2.2.3 Balance symmetry calculation

Balance symmetry was quantified using the Limb Symmetry Index (LSI). For the Prokin system, LSI was calculated from bilateral CoP path-length data obtained during standing. Higher LSI values indicate greater interlimb asymmetry and poorer balance performance (Alshahrani and Reddy, 2026). The LSI was calculated as follows:

$\frac{|(\text{Right}-\text{side value}-\text{Left}-\text{side}-\text{value})|}{(\text{Right}-\text{side value}+\text{Left}-\text{side}-\text{value})/2}\times 100\%.$

### 2.3 Tegner Activity Scale

The Tegner Activity Scale is a validated patient-reported measure of physical activity and sports participation, scored on an 11-point ordinal scale from 0 to 10. A score of 0 indicates severe disability or disease-related sick leave, whereas a score of 10 indicates participation in elite national or international sports. The scale classifies activity by type and records the highest self-reported activity level (Tegner and Lysholm, 1985).

### 2.4 Equipment

#### 2.4.1 fNIRS system

Prefrontal hemodynamic responses were recorded using a continuous-wave fNIRS system (NirScan-6000A, Huichuang Medical Equipment Co., Ltd., Danyang, China) operating at 730 and 850 nm with a sampling rate of 11 Hz. Optodes were secured with an elastic cap positioned according to the international 10–20 EEG system. Adjustable rear straps and forehead padding minimized optode displacement during upper-limb perturbation tasks. Eight light sources and six detectors formed 20 functional channels over the PFC. Channels 35, 36, 43–48, and 51–52 covered the left PFC, whereas channels 33, 34, 37–42, and 49–50 covered the right PFC. Source-detector separation was 3 cm, and embedded 0.8-cm short-separation optode pairs were used to correct superficial noise.

Signal quality control and artifact correction followed established fNIRS best-practice guidelines (Yücel et al., 2025). Channels with raw optical intensity outside 100–9,000 counts or a signal-to-noise ratio < 20 dB were excluded from region-of-interest averaging. Short-separation signals were regressed from corresponding functional channels before calculation of the ΔHbO_2_ integral to reduce extracranial and motion-related confounding. Discrete wavelet transformation was used to remove sharp motion-related spikes, and each trial was normalized to a 30-s quiet-standing baseline to correct slow signal drift. Trials with more than three prominent motion spikes were discarded and repeated.

Oxygenated hemoglobin (ΔHbO₂) was the primary fNIRS outcome (Herold et al., 2018). HbR data were retained during preprocessing for superficial-noise regression but were not included in the formal statistical analyses. NIRSPARK software (v2023) was used for preprocessing. For each participant, repeated trials were averaged within each condition, and signal integrals were calculated for the baseline (−2 to 0 s) and task (0 to 30 s) periods.

#### 2.4.2 sEMG system

Lower-limb neuromuscular activity was recorded using a surface electromyography system (NeuroCare-CT, Nuocheng Electric Co., Ltd., Shanghai, China). The differential preamplifiers had a fixed gain of 1,000×, input impedance > 100 MΩ, and common-mode rejection ratio > 100 dB. Hardware filtering and software processing were used to reduce environmental and movement artifacts and support quantitative analysis of muscle activation and recruitment during balance tasks.

#### 2.4.3 D-WALL system

Visual feedback and motion capture were provided by the D-WALL digital mirror rehabilitation system (TecnoBody S.r.l., Italy). The system includes a 65-inch full-HD display, a 15-inch touchscreen, and a 30-fps markerless infrared three-dimensional camera that tracks 16 body joints. The integrated force platform samples at 100 Hz, with an angular resolution of 0.1̊ and a maximum load of 150 kg. During feedback trials, the screen displayed a real-time three-dimensional skeletal avatar, a blue CoP cursor, a green stability ellipse, and a central cross as the visual target. Data processing and image rendering were performed locally to minimize latency. The D-WALL is a certified medical rehabilitation device compliant with EU Directive 93/42/EEC and the EN 60601 safety standard, and standardized clinical postural-assessment procedures were followed.

### 2.5 Data acquisition

#### 2.5.1 fNIRS acquisition protocol

Participants performed four randomized motor tasks on the dome side of a BOSU ball while prefrontal hemodynamic signals were continuously recorded. Real-time postural feedback was provided by the D-WALL system. Throughout testing, participants wore neutral, thin-soled, flat-heeled training shoes without arch supports, orthotic insoles, or thick cushioning.

**Figure 1:**
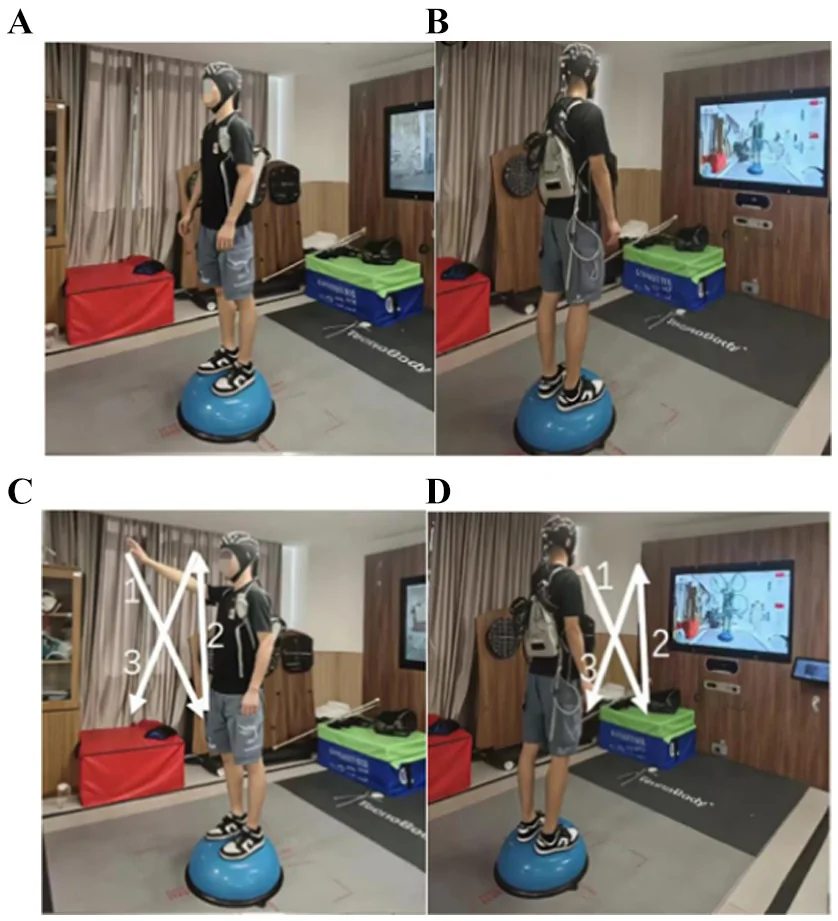
Task conditions. **(A)** BOSU standing without visual feedback. **(B)** BOSU standing with visual feedback. **(C)** BOSU standing with cross-body X-shaped arm movements without visual feedback. **(D)** BOSU standing with cross-body X-shaped arm movements and visual feedback.

Two levels of task difficulty were used. The low-perturbation mode included static BOSU standing without visual feedback (S) (Figure 1A) and with visual feedback (US) (Figure 1B). The high-perturbation dual-task mode included BOSU standing with cross-body X-shaped arm movements without visual feedback (M) (Figure 1C) and with visual feedback (UM) (Figure 1D). To increase postural instability and cognitive–motor load, participants began with the right arm, moving diagonally from the lower right to the upper right and then to the lower left; the left arm then performed the mirrored sequence to complete one X-shaped cycle.

The four conditions were randomized for each participant using the SPSS random-number generator, yielding 24 possible sequences. Each participant completed four to five valid trials per condition. Before testing, participants underwent a 10-15-min familiarization session on the BOSU platform to reduce initial instability and task-related stress.

A 30-s eyes-open quiet-standing baseline was recorded before each task block. Participants stood on a rigid platform under uniform lighting while the D-WALL displayed a blank black screen. Each trial lasted 30 s and was followed by 30 s of rest to minimize fatigue and carryover effects. Total fNIRS recording time was approximately 30 min per participant.

Visual conditions were standardized to isolate feedback-related cortical responses. Screen brightness, background color, and ambient lighting were identical in feedback and non-feedback conditions, and participants kept their eyes open throughout baseline and task periods. Thus, differences in prefrontal hemodynamics were attributed to online corrective feedback rather than nonspecific visual stimulation.

#### 2.5.2 sEMG acquisition protocol

**Figure 2:**
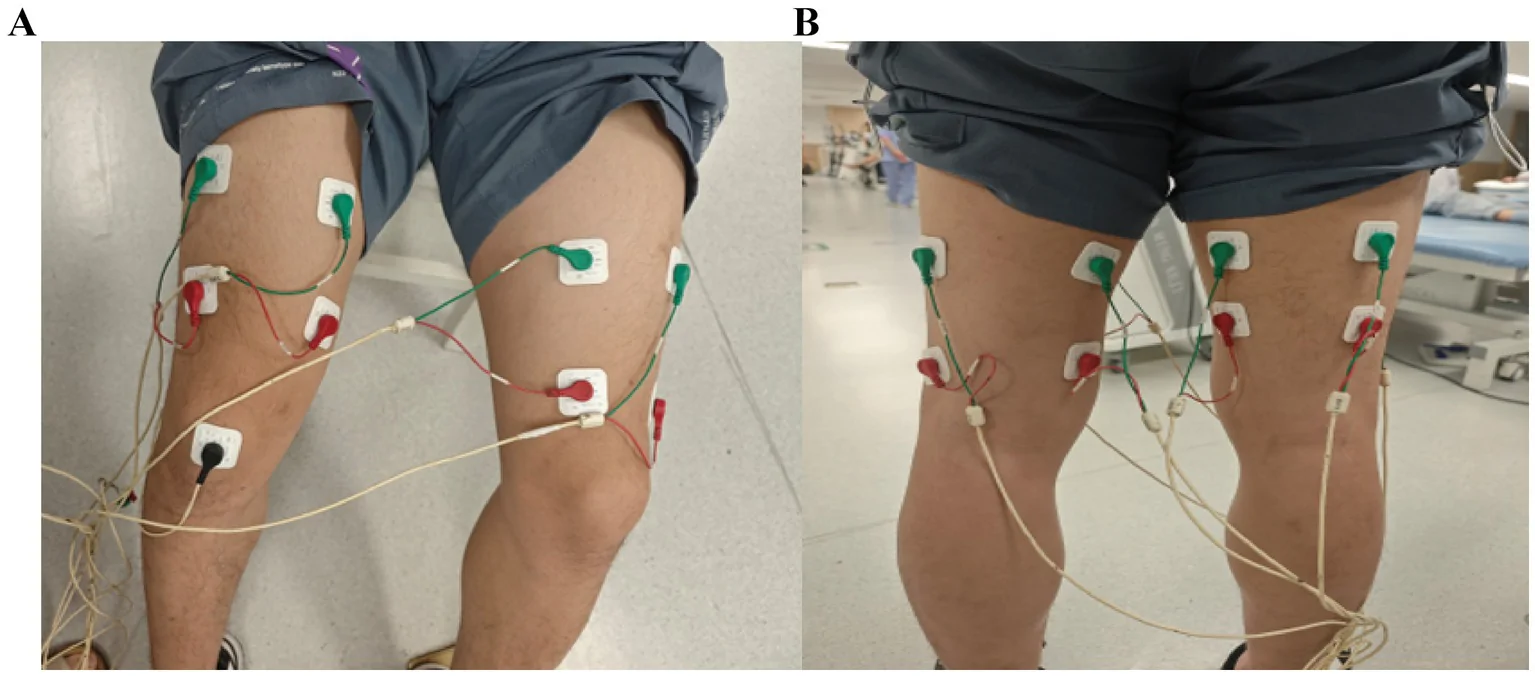
Electrode placement on the lower-limb muscles. **(A)** Electrodes over the bilateral vastus lateralis (VL) and vastus medialis (VM). **(B)** Electrodes over the bilateral biceps femoris (BF) and semitendinosus (ST).

sEMG was recorded concurrently with fNIRS across the four BOSU conditions. The vastus medialis (VM), vastus lateralis (VL), biceps femoris (BF), and semitendinosus (ST) were selected as key thigh muscles contributing to dynamic knee stabilization (Figure 2). The cross-body X-shaped arm task increased whole-body sway and cognitive-motor demand, allowing assessment of knee-muscle recruitment under greater postural challenge.

All acquisition procedures, including electrode placement, skin preparation, filtering, sampling, root mean square (RMS) analysis, and maximal voluntary contraction (MVC) normalization, followed SENIAM guidelines. Disposable bipolar Ag/AgCl electrodes were placed longitudinally over each muscle belly with a 2-cm interelectrode distance, and a reference electrode was placed over the ipsilateral lateral malleolus. Signals were sampled at 2000 Hz, amplified 1,000×, and filtered using a fourth-order Butterworth band-pass filter (10–500 Hz) and a 50-Hz notch filter. RMS amplitude was calculated using nonoverlapping 500-ms windows, and the mean RMS across each 30-s trial was retained for analysis.

MVC was used for normalization. Participants completed three 5-s isometric MVC trials for each muscle in standardized positions, with 60 s of rest between trials and standardized verbal encouragement. The highest RMS value was used as the individual reference, and task-related RMS values were expressed as %MVC. MVC measurements showed excellent within-session reliability (all ICCs > 0.92) (O'Bryan et al., 2024). Three valid trials were obtained for each condition. Normalized signals were then grouped into anterior and posterior thigh muscle groups, and the relative activation proportion of each group was calculated.

### 2.6 Data analysis

#### 2.6.1 Data preprocessing and normality testing

fNIRS and sEMG data were preprocessed using NIRSPARK (v2023) and custom MATLAB scripts, respectively. Valid fNIRS trials were averaged within each condition, and task-related prefrontal ΔHbO_2_ changes were quantified from signal integrals during the baseline (−2 to 0 s) and task (0 to 30 s) periods. Channels were grouped into left and right PFC regions according to the international 10–20 EEG system.

Normality was assessed using the Shapiro–Wilk test for lPFC ΔHbO₂, rPFC ΔHbO₂, and normalized bilateral lower-limb muscle activation ratios. All variables were normally distributed across the four conditions (all *p* > 0.05), supporting the use of repeated-measures analysis of variance (ANOVA) and *post hoc* pairwise comparisons.

*A priori* sample-size calculation was performed in G*Power 3.1 for the repeated-measures design. Based on previous fNIRS balance studies, an effect size of d = 0.62 was assumed. With α = 0.05 and 80% power, the minimum required sample size was 24; the recruited sample of 30 exceeded this requirement.

#### 2.6.2 Statistical analysis

Analyses were performed using SPSS 2019. Participants were stratified by baseline balance performance and habitual physical activity for exploratory subgroup analyses. YBT and Prokin LSI values were dichotomized using prespecified median splits based on all 30 participants. Values at or below the median were assigned to the lower-balance-function group, and values above the median to the higher-balance-function group. Due to tied median values in the dataset, subgroup sample sizes were slightly unbalanced. Pearson correlations using continuous balance indices were also performed to address the loss of information associated with dichotomization. Habitual physical activity was classified using a Tegner score cutoff of 5, which distinguishes low-to-moderate from high activity in healthy young adults (Briggs et al., 2009).

Pearson correlations examined associations among continuous behavioral measures and visual-feedback-induced prefrontal activation separately for static standing and the upper-limb perturbation dual task. Variables included Tegner score, YBT and Prokin LSI values, and the difference in ΔHbO_2_ between feedback and non-feedback conditions. Positive difference values indicated greater prefrontal oxygenation with visual feedback. All correlation tests were two-tailed with a significance level set at α = 0.05.

Three-way mixed repeated-measures ANOVA was used for the primary statistical analyses of prefrontal hemodynamic and neuromuscular outcomes. Subgroup analyses were prespecified exploratory secondary analyses without directional hypotheses. Bonferroni correction was applied to *post hoc* simple-effects comparisons following significant ANOVA results, and decomposed *t* tests were performed only for significant interactions.

The concurrent fNIRS-sEMG analysis was limited to seven participants with low habitual physical activity and marked bilateral balance asymmetry. Because of the small sample, prespecified paired-samples *t* tests were used instead of three-way ANOVA. Bar charts of regional HbO_2_ changes were generated using GraphPad Prism 8.0.

## 3 Results

### 3.1 Participant characteristics and flow diagram

**Figure 3:**
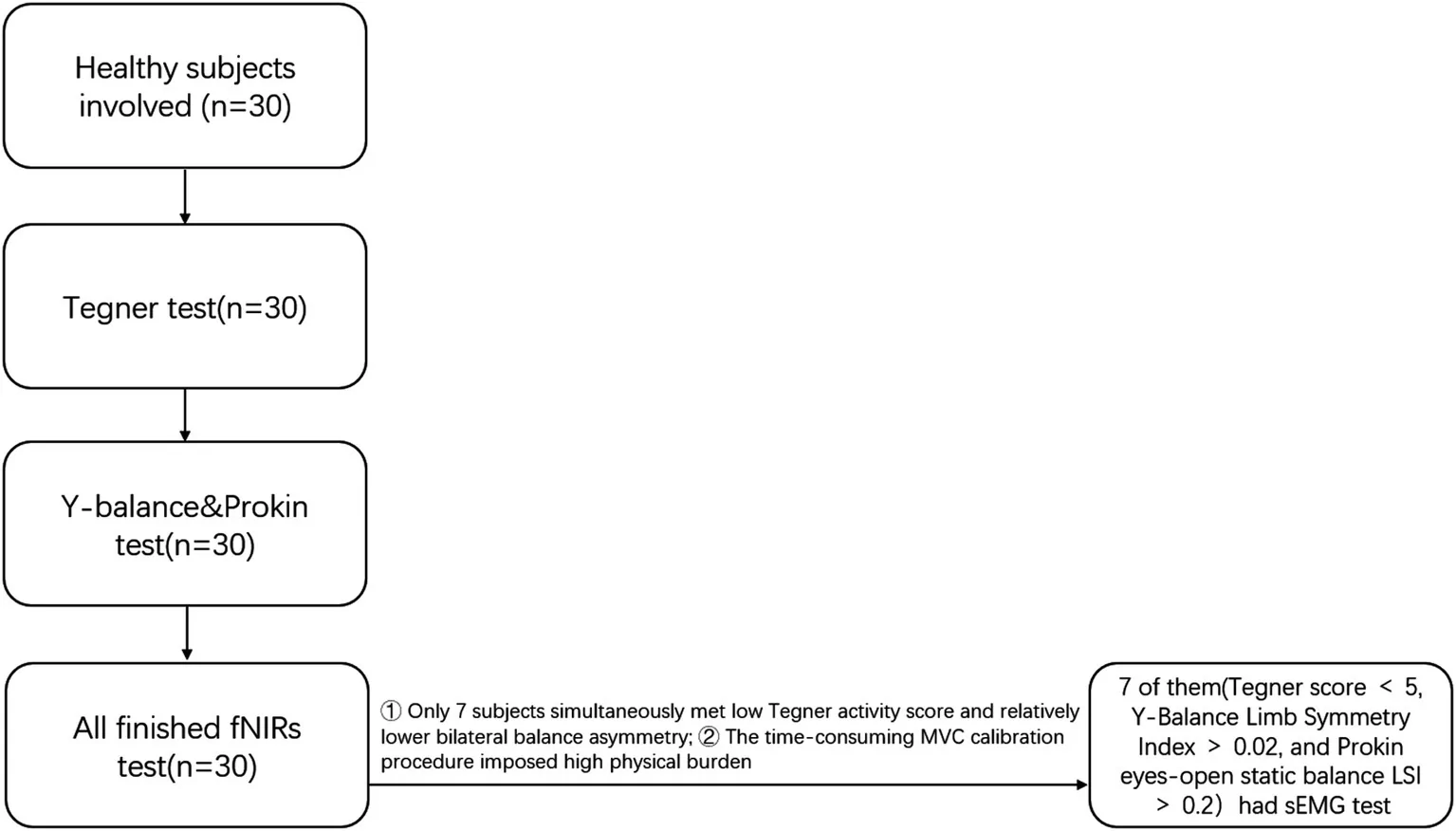
Study flowchart.

Thirty healthy adults (18 men and 12 women) aged 18–40 years were enrolled. Participant characteristics are summarized in Table 1, and the study flow is shown in Figure 3. All 30 participants completed fNIRS testing, whereas 7 completed the supplementary sEMG assessment. Median YBT-LSI, and Prokin-LSI values from the full sample were used as subgroup cutoffs.

**Table 1:** Characteristics of healthy subjects.

Characteristic	HC (*n* = 30)
Age (years, mean ± SD)	27.23 ± 0.82
Sex	
Male, *n* (%)	18 (60%)
Height (cm, mean ± SD)	168.89 ± 5.63
Weight (kg, mean ± SD)	63.79 ± 6.60
Dominant side	
Right, *n* (%)	30 (100%)
Physical activity	
Tegner ≥ 5, *n* (%)	10 (33%)
Tegner < 5, *n* (%)	20 (67%)
Static balance	
Eyes-open path length, LSI > 0.2, *n* (%)	18 (60%)
Eyes-open path length, LSI ≤ 0.2, *n* (%)	12 (40%)
Dynamic balance	
LSI > 0.02, *n* (%)	18 (60%)
LSI ≤ 0.02, *n* (%)	12 (40%)

HC, healthy subjects; LSI, limb symmetry index.

### 3.2 Correlation analysis: static standing

Pearson correlations examined associations among habitual physical activity, static and dynamic balance asymmetry, and visual-feedback-induced changes in bilateral prefrontal ΔHbO_2_ during static standing.

Tegner score was not significantly correlated with balance indices or bilateral prefrontal ΔHbO_2_ differences (all *p* > 0.05). Static balance asymmetry was also not significantly associated with left or right prefrontal ΔHbO_2_ differences (all *p* > 0.05). Dynamic balance asymmetry was moderately and positively correlated with the rPFC ΔHbO_2_ difference (*r* = 0.431, 95% CI [0.062, 0.701], *p* = 0.020), indicating a greater visual-feedback-related increase in rPFC oxygenation among participants with greater interlimb asymmetry. No corresponding association was observed for the lPFC.

### 3.3 Correlation analysis for upper-limb perturbation dual task

Pearson correlations examined associations among Tegner score, static and dynamic balance asymmetry, and visual-feedback-induced prefrontal ΔHbO_2_ differences during the upper-limb perturbation dual task.

Tegner score was not significantly correlated with lPFC or rPFC ΔHbO_2_ differences (all *p* > 0.05). YBT-LSI was moderately and positively correlated with the rPFC ΔHbO_2_ difference (*r* = 0.409, 95% CI [0.033, 0.687], *p* = 0.025). Prokin-LSI also showed a moderate positive trend with the rPFC ΔHbO_2_ difference, but the association was not statistically significant (*r* = 0.350, 95% CI [−0.032, 0.645], *p* = 0.058).

### 3.4 fNIRS outcomes stratified by physical activity

A 2 (visual feedback: feedback vs. no feedback) × 2 (task: static standing vs. upper-limb perturbation dual task) × 2 (habitual physical activity: low vs. high) mixed repeated-measures ANOVA was performed for lPFC ΔHbO_2_. Assumptions were met: Box’s M test indicated homogeneous covariance matrices (*p* = 0.508), sphericity was satisfied, and Levene’s tests showed homogeneous error variances (all *p* > 0.05).

There were no significant main effects of visual feedback (*p* = 0.590), task (*p* = 0.078), or physical activity group (*p* = 0.193). The visual feedback × physical activity interaction was significant (*F* = 5.151, *p* = 0.031, partial η^2^ = 0.155), whereas the other two-way interactions were not (all *p* > 0.05). The three-way interaction was also significant (*F* = 7.612, *p* = 0.010, partial η^2^ = 0.214).

**Figure 4:**
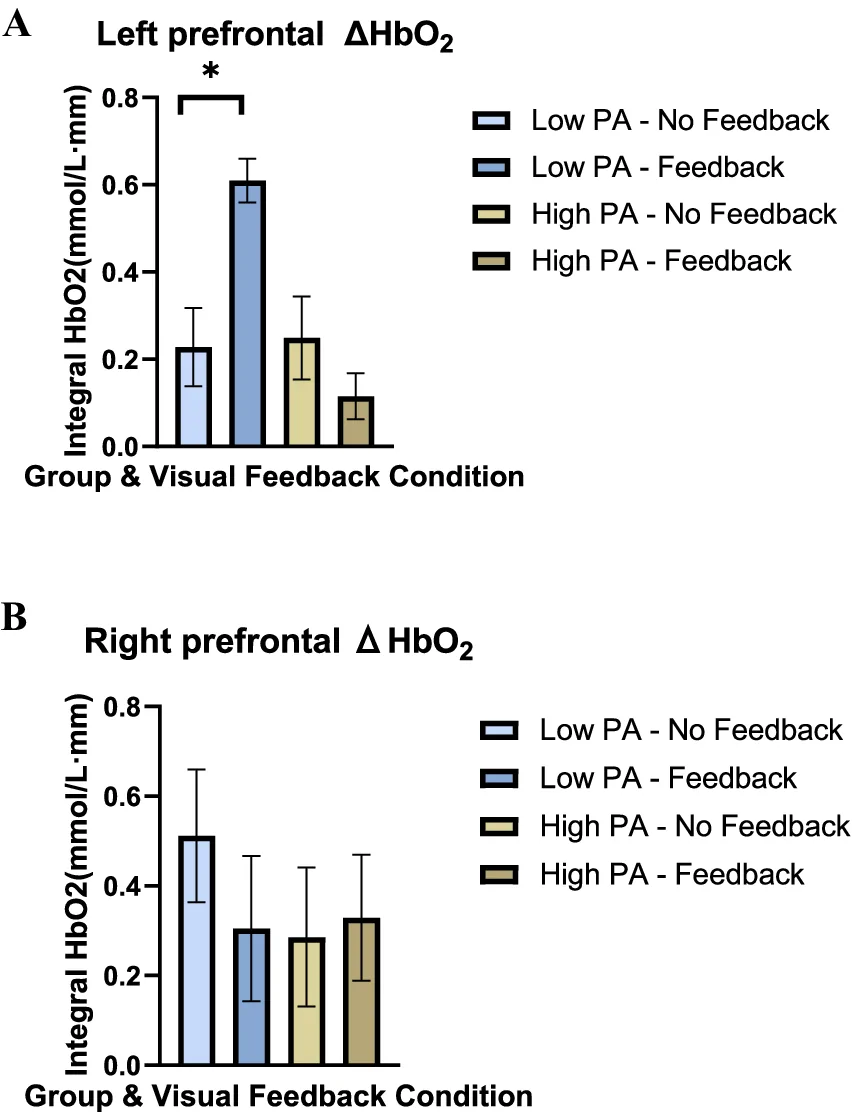
Pooled mean prefrontal ΔHbO₂ across the two postural tasks, stratified by habitual physical activity and visual-feedback condition. **(A)** lPFC ΔHbO₂. The asterisk indicates a significant Bonferroni-corrected simple effect in the low-activity subgroup (*p < 0.05). **(B)** rPFC ΔHbO₂. No simple-effect comparison was significant in either subgroup. Error bars indicate the standard error of the mean (SEM).

Bonferroni-corrected analyses showed significantly higher lPFC ΔHbO_2_ with visual feedback in the low-activity group (mean difference = 0.382, 95% CI [0.003, 0.761], Cohen’s d = 0.57, *p* = 0.049), but not in the high-activity group (mean difference = 0.234, 95% CI [−0.161, 0.629], Cohen’s d = 0.33, *p* = 0.248). Pooled values across tasks are shown in Figure 4A. In the context of the significant three-way interaction, these findings indicate that the lPFC response to visual feedback varied by both activity level and task type.

The same 2 × 2 × 2 mixed repeated-measures ANOVA was performed for rPFC ΔHbO_2_. Model assumptions were met: Box’s M test indicated homogeneous covariance matrices (*p* = 0.724), sphericity was satisfied, and Levene’s tests showed equal error variances (all *p* > 0.05).

There were no significant main effects of visual feedback (*p* = 0.362), task (*p* = 0.360), or physical activity group (*p* = 0.720). The visual feedback × physical activity (*F* = 2.380, *p* = 0.135) and task × physical activity (*F* = 1.155, *p* = 0.292) interactions were not significant. The visual feedback × task interaction was significant (*F* = 8.294, *p* = 0.008, partial η^2^ = 0.235), whereas the three-way interaction was not (*F* = 1.172, *p* = 0.288).

rPFC ΔHbO_2_ was numerically higher with visual feedback in the low-activity group, but the difference was not significant (mean difference = 0.207, 95% CI [−0.031, 0.445], Cohen’s d = 0.40, *p* = 0.087). No difference was observed in the high-activity group (mean difference = 0.043, 95% CI [−0.160, 0.246], Cohen’s d = 0.08, *p* = 0.672). Pooled values are shown in Figure 4B. Thus, rPFC activation varied with the interaction between visual feedback and task, but not with habitual physical activity.

### 3.5 fNIRS outcomes stratified by dynamic balance

A 2 (visual feedback: feedback vs. no feedback) × 2 (task: static standing vs. upper-limb perturbation dual task) × 2 (dynamic balance: poor vs. good) mixed repeated-measures ANOVA was performed for lPFC ΔHbO_2_. Box’s M test indicated homogeneous covariance matrices (*p* = 0.124), and sphericity was satisfied (ε = 1.000). Levene’s tests were nonsignificant for most outcomes; one rPFC ΔHbO_2_ variable in Model 2 violated the homogeneity assumption (*p* = 0.003).

There were no significant main effects of visual feedback (*F* = 0.129, *p* = 0.722, partial η^2^ = 0.005), task (*F* = 2.729, *p* = 0.110, partial η^2^ = 0.089), or dynamic balance group (*F* = 0.907, *p* = 0.349, partial η^2^ = 0.031).

None of the two-way interactions was significant: visual feedback × dynamic balance group (*F* = 2.142, *p* = 0.154, partial η^2^ = 0.071), task × dynamic balance group (*F* = 1.093, *p* = 0.305, partial η^2^ = 0.038), or visual feedback × task (*F* = 0.534, *p* = 0.471, partial η^2^ = 0.019). The three-way interaction was also not significant (*F* = 1.071, *p* = 0.310, partial η^2^ = 0.037).

**Figure 5:**
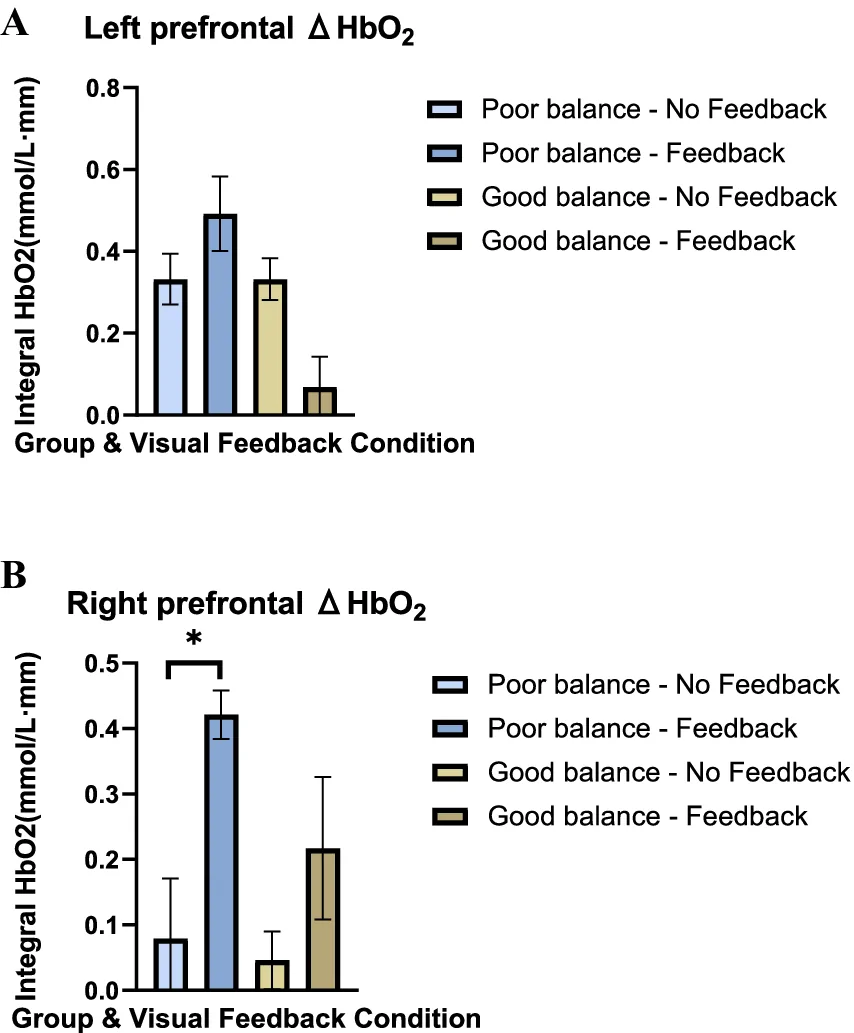
Pooled mean prefrontal ΔHbO₂ across the two postural tasks, stratified by dynamic balance and visual-feedback condition. **(A)** lPFC ΔHbO₂. No Bonferroni-corrected simple effect was significant in either balance subgroup. **(B)** rPFC ΔHbO₂. The asterisk indicates a significant Bonferroni-corrected simple effect in the poor-dynamic-balance subgroup (*p < 0.05). Error bars indicate SEM.

Bonferroni-corrected comparisons showed no difference in lPFC ΔHbO_2_ between feedback and non-feedback conditions in either the poor dynamic balance group (mean difference = 0.160, 95% CI [−0.286, 0.606], Cohen’s d = 0.22, *p* = 0.482) or the good dynamic balance group (mean difference = 0.264, 95% CI [−0.112, 0.640], Cohen’s d = 0.38, *p* = 0.161). Pooled values are shown in Figure 5A. These results provide no evidence that visual feedback, task, or dynamic balance independently or interactively affected lPFC activation.

The same 2 × 2 × 2 mixed repeated-measures ANOVA was performed for rPFC ΔHbO_2_. Box’s M test indicated homogeneous covariance matrices (*p* = 0.493), and sphericity was satisfied (ε = 1.000). Levene’s tests were nonsignificant for most outcomes; one rPFC ΔHbO_2_ variable in Model 2 violated the homogeneity assumption (*p* = 0.022).

There were no significant main effects of visual feedback (*F* = 0.400, *p* = 0.532, partial η^2^ = 0.015), task (*F* = 0.778, *p* = 0.386, partial η^2^ = 0.028), or dynamic balance group (*F* = 0.002, *p* = 0.963, partial η^2^ < 0.001). The task × dynamic balance interaction (*F* = 0.102, *p* = 0.752, partial η^2^ = 0.004) and three-way interaction (*F* = 2.163, *p* = 0.153, partial η^2^ = 0.074) were not significant. Significant interactions were observed for visual feedback × dynamic balance group (*F* = 4.587, *p* = 0.041, partial η^2^ = 0.145) and visual feedback × task (*F* = 6.744, *p* = 0.015, partial η^2^ = 0.200).

Bonferroni-corrected comparisons showed significantly higher rPFC ΔHbO_2_ with visual feedback in the poor dynamic balance group (mean difference = 0.500, 95% CI [0.022, 0.978], Cohen’s d = 0.72, *p* = 0.040), but not in the good dynamic balance group (mean difference = 0.272, 95% CI [−0.280, 0.824], Cohen’s d = 0.31, *p* = 0.333). Pooled values are shown in Figure 5B. Thus, the rPFC response to visual feedback varied with both dynamic balance performance and task difficulty.

### 3.6 fNIRS outcomes stratified by static balance

A 2 (visual feedback: feedback vs. no feedback) × 2 (task: static standing vs. upper-limb perturbation dual task) × 2 (static balance: poor vs. good) mixed repeated-measures ANOVA was performed for lPFC ΔHbO_2_. Model assumptions were met: Box’s M test indicated homogeneous covariance matrices (*p* = 0.197), sphericity was satisfied (ε = 1.000), and Levene’s tests showed homogeneous error variances (all *p* > 0.05).

There were no significant main effects of visual feedback (*F* = 0.342, *p* = 0.563, partial η^2^ = 0.012), task (*F* = 3.686, *p* = 0.065, partial η^2^ = 0.116), or static balance group (*F* = 0.765, *p* = 0.389, partial η^2^ = 0.027). None of the two-way interactions was significant: visual feedback × static balance group (*F* = 0.997, *p* = 0.327, partial η^2^ = 0.034), task × static balance group (*F* = 0.463, *p* = 0.502, partial η^2^ = 0.016), or visual feedback × task (*F* = 0.307, *p* = 0.584, partial η^2^ = 0.011). The three-way interaction was also not significant (*F* = 0.192, *p* = 0.665, partial η^2^ = 0.007).

**Figure 6:**
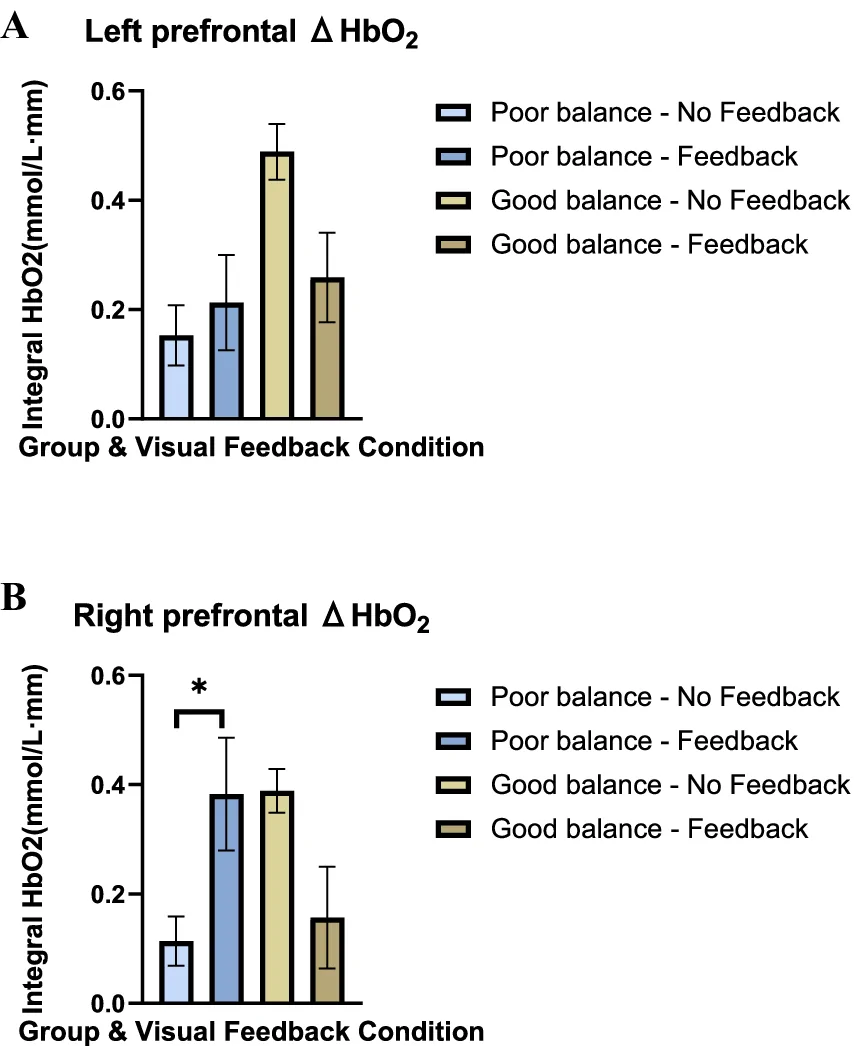
Pooled mean prefrontal ΔHbO₂ across the two postural tasks, stratified by static balance and visual-feedback condition. **(A)** lPFC ΔHbO₂. No Bonferroni-corrected simple effect was significant in either static-balance subgroup. **(B)** rPFC ΔHbO₂. The asterisk indicates a significant Bonferroni-corrected simple effect in the poor-static-balance subgroup (*p < 0.05). Error bars indicate SEM.

Bonferroni-corrected comparisons showed no difference in lPFC ΔHbO_2_ between feedback and non-feedback conditions in either the poor static balance group (mean difference = 0.060, 95% CI [−0.365, 0.485], Cohen’s d = 0.09, *p* = 0.779) or the good static balance group (mean difference = −0.230, 95% CI [−0.627, 0.167], Cohen’s d = −0.34, *p* = 0.256). Pooled values are shown in Figure 6A. No independent or interactive effects on lPFC activation were identified.

The same 2 × 2 × 2 mixed repeated-measures ANOVA was performed for rPFC ΔHbO_2_. Model assumptions were met: Box’s M test indicated homogeneous covariance matrices (*p* = 0.897), sphericity was satisfied (ε = 1.000), and Levene’s tests showed homogeneous error variances (all *p* > 0.05).

There were no significant main effects of visual feedback (*F* = 0.616, *p* = 0.439, partial η^2^ = 0.022), task (*F* = 0.906, *p* = 0.350, partial η^2^ = 0.032), or static balance group (*F* = 0.361, *p* = 0.553, partial η^2^ = 0.013). The task × static balance interaction was not significant (*F* = 0.005, *p* = 0.943, partial η^2^ < 0.001), and the three-way interaction approached but did not reach significance (*F* = 3.890, *p* = 0.059, partial η^2^ = 0.126). Significant interactions were observed for visual feedback × static balance group (*F* = 5.362, *p* = 0.028, partial η^2^ = 0.166) and visual feedback × task (*F* = 7.609, *p* = 0.010, partial η^2^ = 0.220).

Bonferroni-corrected comparisons showed significantly higher rPFC ΔHbO_2_ with visual feedback in the poor static balance group (mean difference = 0.546, 95% CI [0.064, 1.028], Cohen’s d = 0.77, *p* = 0.028), but not in the good static balance group (mean difference = −0.270, 95% CI [−0.806, 0.266], Cohen’s d = −0.33, *p* = 0.312). Pooled values are shown in Figure 6B. Thus, the rPFC response to visual feedback varied with both static balance performance and task difficulty.

### 3.7 Exploratory sEMG and corresponding fNIRS outcomes

An exploratory analysis examined lower-limb muscle coordination in participants with low physical activity and marked bilateral balance asymmetry. Complete sEMG data were available for seven participants who met all criteria: Tegner score < 5, YBT-LSI > 0.02, and Prokin eyes-open static-balance LSI > 0.2.

**Figure 7:**
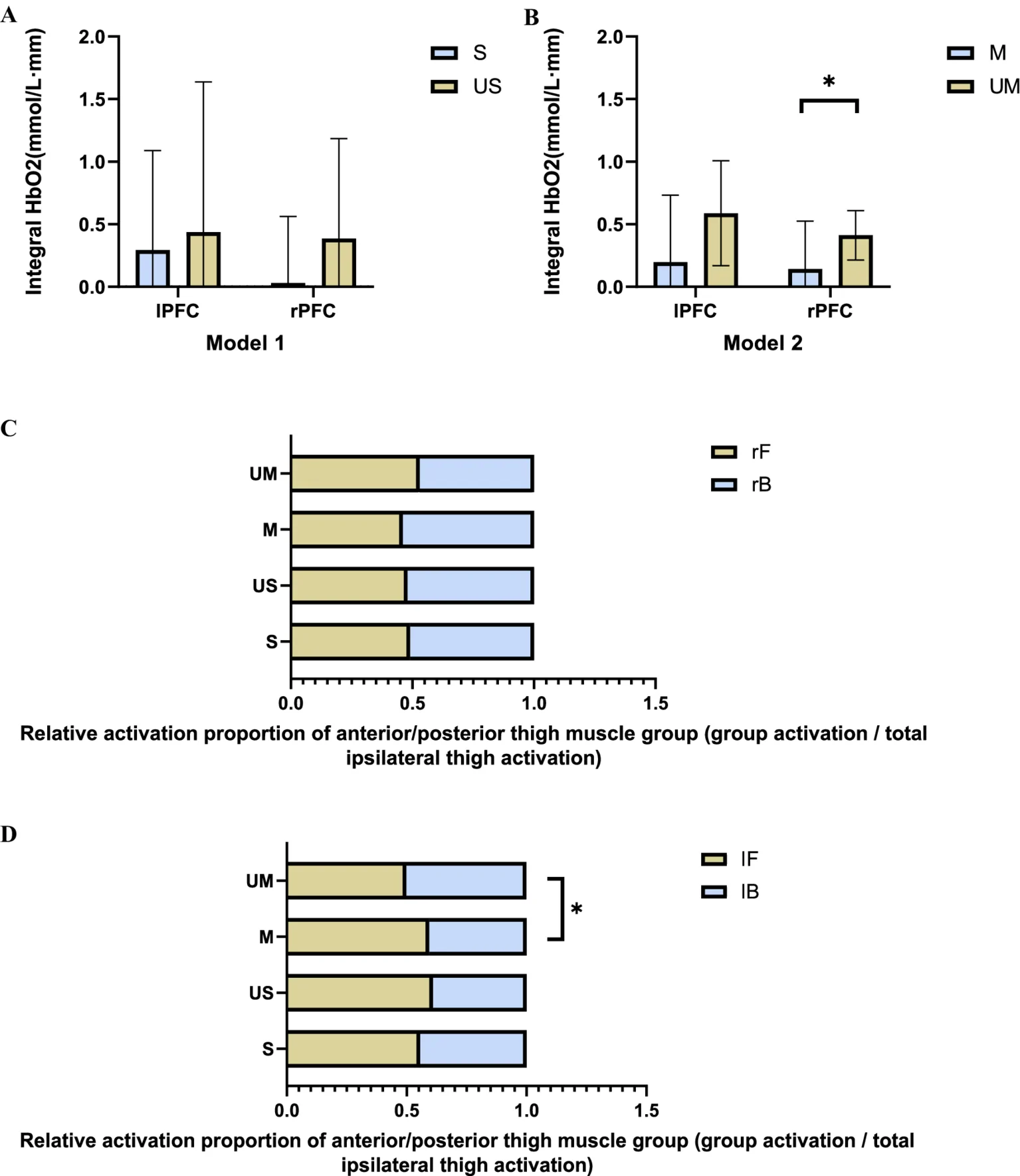
Lower-limb muscle activation proportions and corresponding fNIRS changes across two task models. **(A)** lPFC and rPFC activation in the S and US conditions. **(B)** lPFC and rPFC activation in the M and UM conditions (*p < 0.05); error bars indicate standard deviation (*SD*). **(C)** Relative activation of the right anterior (rF) and posterior (rB) thigh groups. **(D)** Relative activation of the left anterior (lF) and posterior (lB) thigh groups. For panels C and D, relative activation was calculated as the normalized total sEMG of the anterior or posterior thigh group divided by the summed normalized sEMG of the ipsilateral anterior and posterior thigh groups. *p < 0.05.

Relative activation of the anterior and posterior thigh muscle groups was analyzed alongside matched prefrontal fNIRS signals (Figure 7). Each proportion was calculated as the normalized, MVC-scaled sEMG amplitude of one muscle group divided by the summed activation of the ipsilateral anterior and posterior groups; these proportions are plotted on the x-axes of Figures 7C,D.

No significant differences in lPFC or rPFC ΔHbO₂ were observed between the S and US conditions (Figure 7A). Consistent with the full-sample fNIRS results, rPFC ΔHbO₂ was significantly higher in the UM than in the M condition (*t* = 2.85, 95% CI [0.056, 0.780], *p* = 0.029, Cohen’s d = 1.08; Figure 7B).

Relative activation did not differ significantly between the right anterior (rF) and posterior (rB) thigh muscle groups across conditions (*t* = 0.47, 95% CI [−0.128, 0.192], *p* = 0.653, Cohen’s d = 0.18; Figure 7C). In contrast, relative activation of the left posterior thigh group (lB) versus the left anterior thigh group (lF) was significantly higher in the UM than in the M condition (*t* = −2.92, 95% CI [−0.262, −0.032], *p* = 0.013, Cohen’s d = −1.11; Figure 7D). These findings are preliminary because of the small sEMG sample.

## 4 Discussion

This study examined how real-time visual feedback was associated with prefrontal hemodynamics and lower-limb muscle coordination during balance tasks in healthy adults. Three findings were most notable. First, the cortical response to visual feedback differed according to task demand and baseline balance symmetry, with the most consistent effects observed in the rPFC. Second, participants with lower habitual physical activity showed greater lPFC activation under visual feedback. Third, exploratory sEMG data suggested that visual feedback may be accompanied by redistribution of thigh-muscle recruitment. These findings indicate that the neural response to visual feedback is not uniform but may depend on the participant’s motor experience and balance capacity.

### 4.1 Balance function and prefrontal activation across task loads

The balance measure associated with rPFC activation varied with task complexity. During low-perturbation standing, greater dynamic interlimb asymmetry was associated with a larger visual-feedback-related increase in rPFC oxygenation. This pattern may reflect the continuous online correction of body sway in response to D-WALL cues. When cross-body arm movements were added, static asymmetry became the stronger correlate of rPFC recruitment, possibly because the concurrent movement increased cognitive–motor load and the demand for maintaining a stable base of support. Thus, the behavioral feature most closely related to cortical recruitment appeared to shift with the primary postural challenge imposed by the task.

These associations were confined to the rPFC, with no comparable linear relationship in the lPFC. This lateralization is consistent with evidence that the right hemisphere contributes prominently to visuospatial attention and online monitoring of postural errors (Pérennou et al., 1997; Xie et al., 2026). Greater bilateral prefrontal coordination during more demanding tasks may also support the integration of overlapping visual and motor information. Tegner score was not linearly associated with visual-feedback-induced PFC changes, suggesting that immediate cortical recruitment may depend more strongly on current postural demand than on habitual activity considered as a continuous measure. However, the correlation and subgroup analyses were exploratory and should not be interpreted as evidence of distinct neural phenotypes without confirmation in larger samples.

### 4.2 lPFC activation in participants with lower physical activity

Among participants with Tegner scores < 5, lPFC ΔHbO₂ was higher with visual feedback, whereas no significant difference was observed in the higher-activity group. Visual feedback can enhance midfrontal theta synchronization during balance tasks (Chen et al., 2022). These oscillations are thought to support cognitive control by coordinating prefrontal and sensorimotor networks during error monitoring and movement adjustment (Watanabe et al., 2021). Individuals with less habitual motor experience may have less refined internal motor models and may therefore rely more heavily on executive and motor-planning resources when external feedback is available. Visual feedback may also strengthen prefrontal–cerebellar connectivity, facilitating the comparison of expected and observed movement and supporting adaptive motor learning (Nettekoven et al., 2025). This interpretation is plausible but remains speculative because activity level was categorized within a small exploratory sample and the study did not directly measure motor-model quality or network connectivity.

### 4.3 rPFC activation in participants with poorer dynamic balance

Participants with greater dynamic asymmetry showed higher rPFC ΔHbO₂ during visual-feedback dual tasks, whereas those with better dynamic balance showed no consistent change. One possible explanation is error amplification: visual feedback increases the salience of movement deviations and thereby engages midfrontal theta activity involved in detecting postural errors and implementing rapid online corrections (Huang et al., 2017). The rPFC also contributes to inhibitory control and the allocation of attention when actions are uncertain or competing (Hampshire et al., 2009). Participants with poorer dynamic balance may generate more frequent or larger movement errors, increasing the need to suppress ineffective responses and select corrective actions. Accordingly, greater rPFC recruitment may reflect the additional cognitive control required to maintain stability. Nevertheless, the present design did not establish whether increased rPFC activation directly improved task performance or represented a less efficient compensatory response.

### 4.4 rPFC activation in participants with poorer static balance

Visual feedback was also associated with higher rPFC activation in participants with poorer static balance, but not in those with better static balance. This pattern is compatible with a compensatory role of the rPFC when postural control is challenging (Huang et al., 2017; Hampshire et al., 2009). Increased rPFC activation has been reported as a compensatory response during motor tasks in patients with stroke (Park et al., 2021), although evidence from patients with cortical lesions should be extrapolated cautiously to healthy adults. In the present sample, greater activation may have supported attention to visual error signals or compensated for less efficient recruitment of sensorimotor regions. Because performance improvement was not directly linked to the hemodynamic response, increased activation should not automatically be interpreted as more effective control.

### 4.5 Prefrontal activation and lower-limb muscle coordination

In the exploratory sEMG subgroup (*n* = 7), higher rPFC ΔHbO₂ with visual feedback was accompanied by greater relative activation of the left posterior thigh muscles. Visual cues may partially compensate for less reliable proprioceptive input or multisensory integration, increasing reliance on visually guided descending motor control (Bogdanov et al., 2018). Posterior thigh muscles contribute to antigravity support, stabilization of the hip and knee, and adjustments of the base of support. Their recruitment may therefore increase when participants attempt to match a continuously displayed postural target (Sayenko et al., 2010; Nam and Lee, 2022). Visual feedback may also encourage combined ankle-hip strategies, in which the hamstrings contribute to hip-based corrections. The greater response in the left limb is consistent with evidence that the nondominant leg may play a greater stabilizing role during balance tasks (Schorderet et al., 2021). However, the relative activation measure does not establish absolute increases in muscle force, and the very small sample makes the finding vulnerable to sampling variability. The sEMG result should therefore be viewed only as hypothesis-generating evidence for a possible link between prefrontal recruitment and redistribution of lower-limb muscle coordination.

Increased PFC ΔHbO₂ should not be interpreted exclusively as beneficial compensation or optimized motor planning. Higher prefrontal oxygenation may also reflect greater cognitive load, sustained attentional monitoring, or reduced automaticity during visually guided balance (Sayenko et al., 2010). Participants with lower activity or poorer balance may need to compare ongoing postural deviations continuously with the visual target, increasing conscious supervision of movements that might otherwise be regulated more automatically. Compensatory motor control and attentional load are not mutually exclusive and may have contributed simultaneously to the observed responses. Future studies should independently manipulate feedback complexity, cognitive load, and motor adaptation and should relate cortical activation to objective improvements in postural performance. Such designs would help determine whether greater PFC recruitment represents effective adaptation, increased task difficulty, or both.

### 4.6 Limitations

This study has several limitations. First, its cross-sectional design identifies associations but cannot establish causal relationships among visual feedback, cortical hemodynamics, and muscle activation. Second, subgroup sizes were small (10–20 participants), and complete sEMG data were available for only seven participants. The sEMG comparisons were therefore underpowered, and all neuromuscular findings should be considered preliminary. Third, median-split dichotomization reduced information contained in the continuous activity and balance measures and may have decreased statistical power. Although complementary correlation analyses were performed, multiple exploratory subgroup models and *post hoc* comparisons increased the risk of Type I error despite Bonferroni correction. The observed subgroup patterns require independent confirmation using larger samples and prespecified directional hypotheses. Fourth, all participants were healthy young adults, limiting generalizability to older adults and patients with clinically relevant balance impairment. Finally, the 10–15-min familiarization period may have introduced habituation or sensorimotor learning that influenced baseline PFC activation. Future work should use adequately powered prospective or randomized designs, include clinical populations, quantify changes in balance performance alongside cortical responses, and use counterbalanced familiarization protocols to separate training, habituation, and feedback effects.

## 5 Conclusion

Visual feedback was associated with altered prefrontal cortical activation and lower-limb muscle coordination during balance tasks in healthy adults. The observed responses varied according to habitual physical activity, balance symmetry, and task demands, with exploratory evidence of corresponding changes in posterior thigh muscle recruitment. These findings support further investigation of visual-feedback-based balance training, but clinical effectiveness cannot be inferred from this healthy sample. Larger prospective studies in individuals with impaired postural control are needed.

## Data Availability

The raw data supporting the conclusions of this article will be made available by the authors, without undue reservation.

## Glossary

fNIRS: Functional near-infrared spectroscopy
sEMG: Surface electromyography
lPFC: Left prefrontal cortex
rPFC: Right prefrontal cortex
PET: Positron emission tomography
fMRI: Functional magnetic resonance imaging
EEG: Electroencephalography
HbO₂: Oxygenated hemoglobin
HbR: Deoxygenated hemoglobin
PFC: Prefrontal cortex
CoP: Center of pressure
YBT: Y-Balance Test
LSI: Limb Symmetry Index
PRO: Patient-reported outcome
RMS: Root mean square
MVC: Maximal voluntary contraction
VM: Vastus medialis
VL: Vastus lateralis
BF: Biceps femoris
ST: Semitendinosus
ROI: Region of interest
